# The Orphan Nuclear Receptor NR4A1 Protects Pancreatic β-Cells from Endoplasmic Reticulum (ER) Stress-mediated Apoptosis*

**DOI:** 10.1074/jbc.M115.654863

**Published:** 2015-07-08

**Authors:** Cong Yu, Shang Cui, Chen Zong, Weina Gao, Tongfu Xu, Peng Gao, Jicui Chen, Dandan Qin, Qingbo Guan, Yuantao Liu, Yuchang Fu, Xia Li, Xiangdong Wang

**Affiliations:** From the ‡ The Department of Cell Biology, Shandong University School of Medicine, Jinan, China, 250012,; § The Department of Endocrinology, Provincial Hospital affiliated to Shandong University, Jinan, China, 250021,; ¶ Department of Endocrinology, Qingdao Municipal Hospital, Qingdao, China, 266071,; ‖ The Department of Nutrition Sciences, University of Alabama at Birmingham, Alabama 35294, and; ** Key Laboratory of Protein Sciences for Chronic Degenerative Diseases in Universities of Shandong (Shandong University), Jinan, China 250012

**Keywords:** apoptosis, beta cell (B-cell), endoplasmic reticulum stress (ER stress), pancreatic islet, survivin, NR4A1

## Abstract

The role of NR4A1 in apoptosis is controversial. Pancreatic β-cells often face endoplasmic reticulum (ER) stress under adverse conditions such as high free fatty acid (FFA) concentrations and sustained hyperglycemia. Severe ER stress results in β-cell apoptosis. The aim of this study was to analyze the role of NR4A1 in ER stress-mediated β-cell apoptosis and to characterize the related mechanisms. We confirmed that upon treatment with the ER stress inducers thapsigargin (TG) or palmitic acid (PA), the mRNA and protein levels of NR4A1 rapidly increased in both MIN6 cells and mouse islets. NR4A1 overexpression in MIN6 cells conferred resistance to cell loss induced by TG or PA, as assessed by MTT (3-(4,5-dimethylthiazol-2-yl)-2,5-diphenyltetrazolium bromide) assay, and TUNEL assays indicated that NR4A1 overexpression also protected against ER stress-induced apoptosis. This conclusion was further confirmed by experiments exploiting siRNA to knockdown NR4A1 expression in MIN6 cells or exploiting NR4A1 knock-out mice. NR4A1 overexpression in MIN6 cells reduced C/EBP homologous protein (CHOP) expression and Caspase3 activation induced by TG or PA. NR4A1 overexpression in MIN6 cells or mouse islets resulted in Survivin up-regulation. A critical regulatory element was identified in Survivin promoter (−1872 bp to −1866 bp) with a putative NR4A1 binding site; ChIP assays demonstrated that NR4A1 physically associates with the Survivin promoter. In conclusion, NR4A1 protects pancreatic β-cells against ER stress-mediated apoptosis by up-regulating Survivin expression and down-regulating CHOP expression, which we termed as “positive and negative regulation.”

## Teaser

**Background:** It is not known whether NR4A1 plays a role in ER stress-induced β-cell apoptosis.

**Results:** NR4A1 expression in β-cells or islets correlated positively with Survivin expression and negatively with CHOP expression and apoptosis rate upon treatment with ER stress inducers.

**Conclusion:** NR4A1 protects β-cells from ER stress-induced apoptosis by up-regulating Survivin and down-regulating CHOP expression.

**Significance:** Our findings provide clues to prevent diabetes.

## Introduction

The occurrence and development of type 2 diabetes mellitus is closely related to the loss of islet β-cells and their function (1). Pancreatic β-cells are sensitive to sustained hyperglycemia or high concentrations of fat, which can change the Ca^2+^ concentration in pancreatic β-cells and induce ER stress, leading to apoptosis (2, 3). When the ER protein-folding machinery fails to process unfolded protein, the cell enters a state of ER stress. ER stress induces the unfolded protein response (UPR),^3^ which is a feedback mechanism to eliminate adverse conditions or to adjust the intracellular homeostasis. Increasing evidence suggests that many pathological conditions continuously disrupt ER homeostasis and lead to a long term chronic UPR, which can cause many diseases (4). Pancreatic β-cells and their activity are essential for glucose and metabolic homeostasis.

NR4A1, also known as Nur77, belongs to the NR4A subfamily, which also includes NR4A2 (Nurr1) and NR4A3 (NOR-1, MINOR), is an orphan nuclear receptor with an unknown natural ligand. NR4A subfamily members primarily function by modulating gene expression upon exogenous stimulation. Many stimuli, including nerve growth factor (NGF), tumor necrosis factor (TNF), serum, thapsigargin (TG), free fatty acids (FFA), 12-*O*-tetradecanoylphorbol 13-acetate, and CD437, have been shown to induce NR4A family member transcription in many cell types (5–11). Recent studies have indicated that NR4A1 is involved in apoptosis in many cell lines. When cells were stimulated with apoptosis-inducing agents such as CD437 or 12-*O*-tetradecanoylphorbol 13-acetate, NR4A1 translocated from the nucleus to the cytoplasm, where it interacted with the mitochondrial membrane, resulting in cytochrome *c* release, thereby initiating the apoptotic process (12–18). However, other studies have demonstrated that NR4A1 localized to the nucleus upon EGF stimulation and induced the transcription of downstream genes and cell proliferation but did not induce apoptosis (19). Therefore, in this study we aimed to determine whether NR4A1 acts as an anti-stress factor to protect β-cells against ER stress-induced apoptosis. If NR4A1 protects pancreatic β-cells from apoptosis, it is important to understand the underlying mechanism.

## Experimental Procedures

### 

#### 

##### Reagents and Cell Culture

Cell culture medium and fetal bovine serum were purchased from Hyclone (Thermo Fisher Scientific Inc., Bremen, Germany). All restriction endonucleases were purchased from New England BioLabs Inc. (Beijing, China). MTT, thapsigargin, and sodium palmitate were purchased from Sigma. Puromycin was purchased from InvivoGen. Wild-type and NR4A1 knock-out mice were purchased from The Jackson Laboratory and were fed *ad libitum* and maintained on a specific pathogen free animal facility with individual ventilated caging system under 12-h light/dark cycles. MIN6 cells were cultured as previously described (20). Cells were incubated overnight and then treated with various agents.

##### Mouse Islet Separation and Purification

Mouse pancreatic islets were isolated from adult C57BL/6J mice after ductal distension of the pancreas and digestion of the tissue with collagenase P (Roche Applied Science) and density gradient centrifugation with Histopaque 1077 (Sigma) according to the classic method with modifications described elsewhere (21, 22).

##### Immunofluorescence Staining

Mouse islets were prepared as described above and fixed with 3.7% paraformaldehyde for 30 min, then penetrated by 0.5% Triton X-100 in PBS for 2 min on ice. After washing with PBS 3 times, the islets were incubated with goat serum for 1 h and thereafter incubated with rabbit anti-NR4A1 antibody (Santa Cruz) and guinea pig anti-insulin antibody (Dako, Produktionsvej, Denmark) at 4 °C overnight. After washing with PBS three times, the islets were incubated with secondary antibodies conjugated to Alexa 488 and 594 (Invitrogen), respectively for 1 h. Then the islets were stained with DAPI for 5 min and mounted on glass slides after washing with PBS. The pictures were taken under a 10× objective lens on a Nikon microscope at the same instrument setting for each kind of staining.

##### Plasmid Construction

Mouse cDNAs were obtained from MIN6 cells, and mouse genomic DNAs were obtained from C57BL/6J liver. We amplified the NR4A1 cDNA using a pair of primers based on the NR4A1 gene sequence (Gene ID 15370 database, Genome) and then cloned the cDNA into the LV5 lenti-vector expressing GFP from GenePharma Co., Ltd (Shanghai, China). The Survivin promoter reporter (−2000 bp) was amplified from mouse genomic DNA (Gene ID 11799 database, Genome) by PCR using a pair of primers and cloned into the pGL3 luciferase reporter vector (Promega) to generate pGL3-Survivin. PGL3-NF-κB (Gene ID 18033 database, Genome), pGL3-Bcl-2 (Gene ID 12043 database, Genome), and the Survivin promoter reporters of different lengths (-1865 bp, -1500 bp, -500 bp, -100 bp) were prepared in the same way as mentioned above. All cloned DNA fragments were sequenced to confirm the correct sequences by Sangon Biotech Co., Ltd. (Shanghai, China).

##### Lentiviral Infection and Stable Cell Line Selection

Lentivirus encoding full-length NR4A1 and control lentivirus were generated by GenePharma. MIN6 cells were infected with recombinant NR4A1 lentiviral stocks or control lentiviral stocks, and stable cell clones were selected under puromycin selection. Western blotting analysis was used to select NR4A1 overexpression cell lines (designated as OV) and control cell lines (designated as NC). NR4A1 knockdown cell colonies were also generated with lentivirus encoding siRNA specific for NR4A1 in the same way as described above. The NR4A1 knockdown cell line was designated as KD, and the control cell line was designated as CON.

##### Dual Luciferase Reporter Assays

NC and OV cells were seeded into 24-well plates for 24 h before transfection. Transient transfection of MIN6 cells with reporter plasmids plus an internal control plasmid vector (pRL-TK) was accomplished with Turbofect transfection reagent (Thermo Scientific). At 24 h post-transfection, luciferase activity was measured by using a Dual Luciferase reporter assay kit (Beyotime Institute of Biotechnology, Shanghai, China) according to the manufacturer' s instructions. The experiments were performed in triplicate and repeated at least three times.

##### Chromatin Immunoprecipitation (ChIP) Analysis

ChIP analysis was accomplished using a ChIP Assay kit (Beyotime) according to manufacturer's instructions. After adenoviral infection with Ad-GFP or Ad-NR4A1-HA for 48 h, MIN6 cells were fixed with 1% formaldehyde for 10 min at 37 °C. An anti-HA monoclonal antibody was applied for immunoprecipitation. A pair of primers, 5′-CCCGAAACAAATCCCACATACAAGT-3′ (forward) and 5′-ACAAAAGGAAAAAACCCTAGTCAGG-3′ (reverse), was designed to amplify the specific target sequence (−1942 bp to −1711 bp) of the Survivin promoter.

##### Recombinant Adenovirus Generation and Infection

The AdEasy System was exploited to generate recombinant adenovirus expressing NR4A1-HA and GFP (designated as Ad-NR4A1). The control virus was generated in the same way, but it expressed only GFP (designated as Ad-GFP). Two adenoviruses (Ad-NR4A1 and Ad-GFP) were amplified, purified, and enriched according to standard techniques (23).

##### MTT Assay

MIN6 cells were seeded in 96-well plates and after 24 h were incubated with fresh medium containing TG, PA, or vehicle for various lengths of time. A routine MTT assay was then completed, and the absorbance values were measured using a microplate reader (Bio-Rad) at 570 nm.

##### TUNEL Assay

OV or NC cells were seeded on coverslips with fresh medium, grown overnight in 24-well plates, and then incubated with TG for 24 h. TUNEL staining was performed with a one-step TUNEL apoptosis assay kit (Beyotime) according to the manufacturer's instructions.

##### Reverse Transcription PCR and Quantitative Real-time PCR Assay

Total RNA was purified from mouse islets using an RNeasy Mini kit (Qiagen). Total RNA isolation from cultured cells, reverse transcription PCR, and quantitative real-time PCR (qPCR) were performed as previously described (20). Relative gene expression was calculated by the ΔΔCq method (24). All experiments were performed at least in triplicate. The primers used are listed in Table 1. tXBP1 represents total XBP1; sXBP1 represents spliced XBP1.

**TABLE 1:** **Primers used for PCR**

Primer	Sequence
NR4A1 (+)	5′-ATGCCTCCCCTACCAATCTTC-3′
NR4A1 (−)	5′-CACCAGTTCCTGGAACTTGGA-3′
Chop (+)	5′-CTGGAAGCCTGGTATGAGGAT-3′
Chop (−)	5′-CAGGGTCAAGAGTAGTGAAGGT-3′
Bip (+)	5′-ACTTGGGGACCACCTATTCCT-3′
Bip (−)	5′-ATCGCCAATCAGACGCTCC-3′
tXBP1 (+)	5′-TGGCCGGGTCTGCTGAGTCCG-3′
tXBP1 (−)	5′-GTCCATGGGAAGATGTTCTGG-3′
sXBP1 (+)	5′-CTGAGTCCGAATCAGGTGCAG-3′
sXBP1 (−)	5′-GTCCATGGGAAGATGTTCTGG-3′
Bcl-2 (+)	5′-ATGCCTTTGTGGAACTATATGGC-3′
Bcl-2 (−)	5′-GGTATGCACCCAGAGTGATGC-3′
Survivin (+)	5′-GAGGCTGGCTTCATCCACTG-3′
Survivin (−)	5′-ATGCTCCTCTATCGGGTTGTC-3′
Gadd34 (+)	5′-GAGGGACGCCCACAACTTC-3′
Gadd34 (−)	5′-TTACCAGAGACAGGGGTAGGT-3′
PP1α (+)	5′-ATGACCTTCTACGGCTGTTTG-3′
PP1α (−)	5′-TGCCCCGATCTACATAATCCC-3′
18S rRNA (+)	5′-CGCGGTTCTATTTTGTTGGT-3′
18S rRNA (−)	5′-AGTCGGCATCGTTTATGGTC-3′

##### Separation and Extraction of Nuclear and Cytoplasmic Proteins

Cells were washed with ice-cold PBS and scraped from the wells with the plates on ice. A nuclear and cytoplasmic protein extraction kit (Beyotime) was applied to separate these two cellular components according to the manufacturer's instructions.

##### Western Blotting Analysis

Proteins were isolated and analyzed according to previously described methods (20, 25). The primary antibodies used were as follows: NR4A1, Survivin (Proteintech, Wuhan, China), phospho-eIF2α (Ser-51), eIF2α, CHOP (Cell Signaling Technology, Boston, MA), Caspase3–17-kDa, Bcl-2, Lamin A (Bioworld Technology, Louis Park, MA), and growth arrest and DNA damage-inducible protein-34 (GADD34; Biosynthesis Biotechnology Co., Beijing, China). Secondary antibodies conjugated to HRP (Zhongshan Golden Bridge Biotechnology, Beijing, China) were used for Western blotting.

##### Statistical Analysis

The statistical data are presented as the mean ± S.E. The statistical analysis of differences was performed using the two-tailed Student's *t* test or with analysis of variance in GraphPad Prism 5.0. *p* < 0.05 (*) was considered significant, and *p* < 0.01 (**) was considered very significant.

## Results

### 

#### 

##### NR4A1 Gene Expression Is Induced in MIN6 Cells upon TG or PA Treatment

**FIGURE 1.**
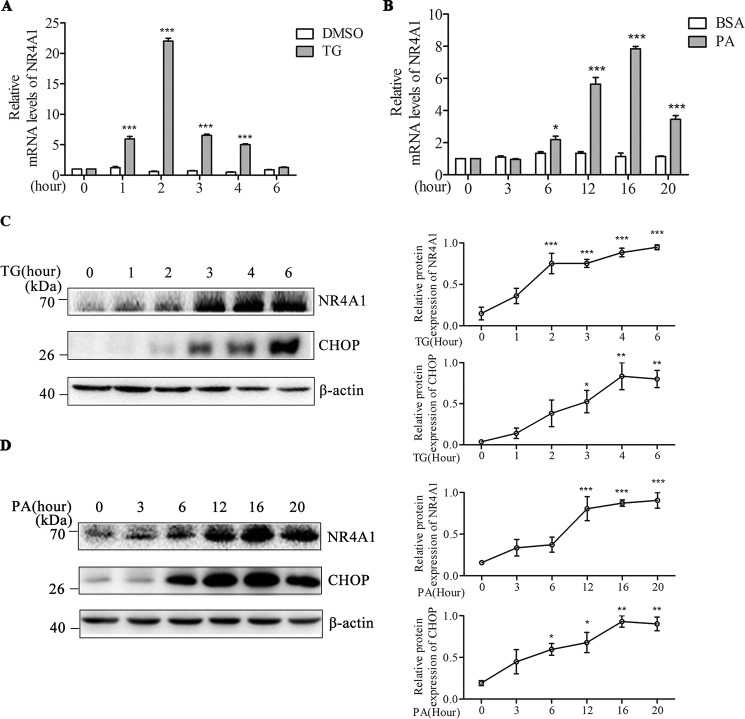
**Analysis of the effect of TG or PA on NR4A1 expression in MIN6 cells.** *A* and *B*, relative NR4A1 mRNA levels in response to 0.5 μm TG (*A*) (DMSO as control) or 0.4 mm PA (*B*) (BSA as control) at various time points, as determined by qPCR. *C* and *D*, protein expression of NR4A1 and CHOP in response to 0.5 μm TG (*C*) and 0.4 mm PA (*D*) at various time points, assessed by Western blotting. Densitometric analyses of the blots are shown as curves. The data show the means of three independent experiments, *p < 0.05, ***p < 0.001 *versus* the DMSO or BSA control.

We examined NR4A1 mRNA expression in MIN6 cells treated with TG (0.5 μm) or PA (0.4 mm) over several time points and found that TG treatment induced NR4A1 mRNA expression, which reached its peak at 2 h (Fig. 1*A*). PA also induced NR4A1 mRNA expression, with a peak at 16 h (Fig. 1*B*). We also examined NR4A1 protein expression after TG or PA treatment and found that treatment with either TG or PA increased NR4A1 protein expression in MIN6 cells. CHOP is regarded as a marker of TG- or PA-induced ER stress, and we found that both inducers increased CHOP expression (Fig. 1, *C* and *D*). These data suggest that inducers of ER stress can induce NR4A1 expression in MIN6 cells.

##### NR4A1 Expression Is Induced in Mouse Islets with TG Treatment

**FIGURE 2.**
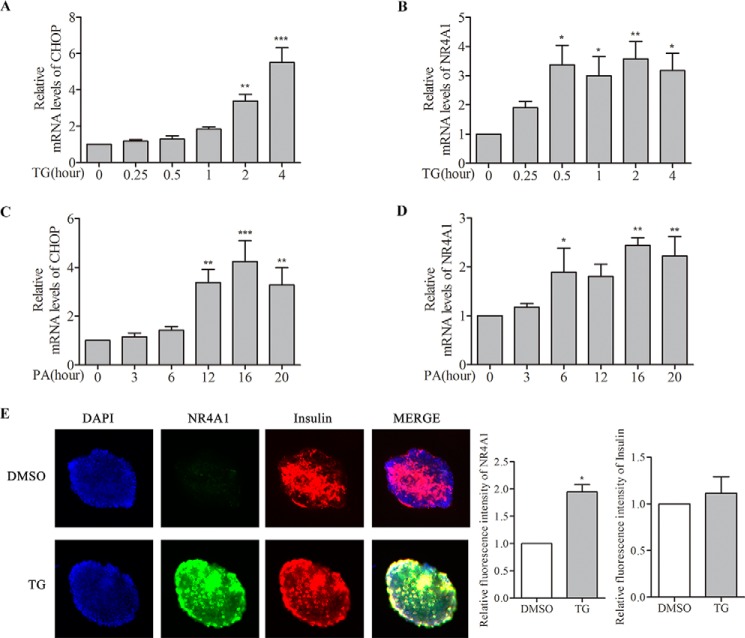
**Analysis of the effect of TG or PA on NR4A1 expression in mouse islets.** Mouse islets were isolated from C57BL/6J mice, and total RNA of mouse islets was prepared using an RNeasy Mini Kit. *A–D*, relative mRNA levels of CHOP and NR4A1 in response to 0.5 μm TG (*A* and *B*) or 0.4 mm PA (*C* and *D*) at various time points were determined by qPCR. *E*, determination of the induced NR4A1 expression in pancreatic β-cells upon TG or PA treatment. Mouse islets were treated with 0.5 μm TG for 6 h, and double immunofluorescence staining was performed with anti-insulin and anti-NR4A1 antibodies from different species. The *top panel* is an islet treated with DMSO as a control, and the *lower panel* is an islet treated with TG. *Blue* represents DAPI, *green* represents NR4A1, *red* represents insulin, and *MERGE* of the three colors. The histograms indicate relative fluorescence intensity (=total red densitometry value/islet surface area). The data show the means of three independent experiments, *, *p* < 0.05; **, *p* < 0.01; ***, *p* < 0.001 *versus* 0 h or DMSO control.

To investigate whether ER stress-inducing reagents increase NR4A1 expression in mouse islets, we isolated islet cells from 16-week-old C57BL/6J mice. The isolated mice islets were treated with TG (0.5 μm) or PA (0.4 mm) for different time points. We found that TG and PA treatment induced a time-dependent rise in mRNA for sXBP1, Bip (immunoglobulin-binding protein) (data not shown), and CHOP (Fig. 2, *A* and *C*), which are markers for ER stress. As expected, TG or PA treatment also increased the NR4A1 mRNA level in a time-dependent manner in purified mouse islets (Fig. 2, *B* and *D*). TG- or PA-induced NR4A1 mRNA expression peaked at earlier time points than that of CHOP (Fig. 2, *A–D*). Olivier Briand (26) previously also showed that PA induces NR4A1 expression in mouse islet cells. To confirm if the increased NR4A1 expression induced by TG and PA in mouse islets occurred in β-cells, we did double immunofluorescence staining with specific antibodies against NR4A1 and Insulin. The images (Fig. 2*E*) showed that TG treatment increase NR4A1 expression (*green*), and it was mostly co-localized with insulin (*red*) in islets. These results confirmed that the two ER stress inducers truly induce NR4A1 expression in β-cells of mouse islets.

##### NR4A1 Expression in MIN6 Cells Confers Resistance to TG- or PA-induced Apoptosis

**FIGURE 3.**
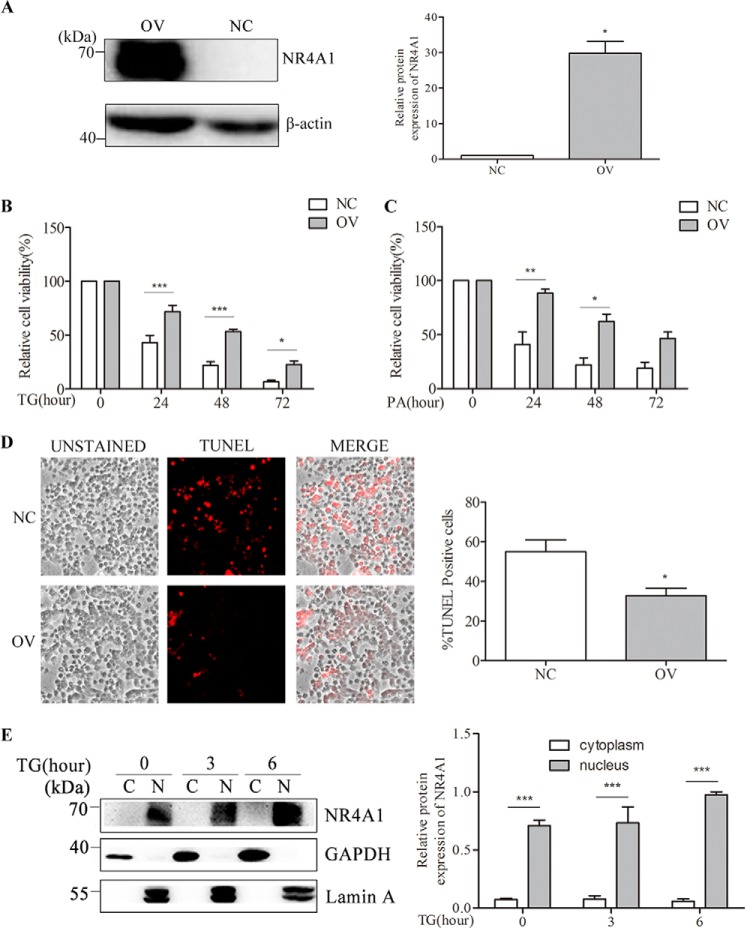
**The role of NR4A1 on β-cell apoptosis.** *A*, Western blotting verification of MIN6 cell lines stably overexpressing NR4A1 (designated as OV) and normal control clones (designated as NC). *B* and *C*, NC and OV MIN6 cell viability upon TG treatment (*B*) or PA treatment (*C*) for various time periods was analyzed using MTT assays. *D*, NC and OV cells were treated with 0.5 μm TG for 24 h. Then the percentage of apoptotic cells was analyzed by TUNEL staining. In this representative image, the cells stained *red* are apoptotic. *E*, the localization analysis of NR4A1 in OV cells. OV cells were treated with 0.5 μm TG for various lengths of time. Nuclear (*N*) and cytoplasmic (*C*) proteins were separated with a protein extraction kit as described under “Experimental Procedures.” GAPDH is a marker for the cytoplasm, and lamin A is a marker for the nucleus. Densitometric analyses of the Western blots and cell death rate are shown as histograms. The data show the means of three independent experiments. *, *p* < 0.05; **, *p* < 0.01; ***, *p* < 0.001 *versus* NC.

It is well known that sustained ER stress can ultimately lead to apoptosis. To investigate whether NR4A1 plays a role in ER stress-induced apoptosis in MIN6 cells, we constructed a lentiviral vector encoding NR4A1 in which both NR4A1 and GFP are expressed. We infected MIN6 cells with the NR4A1-expressing lentivirus or control lentivirus (lentivirus only expressing GFP) and obtained several NR4A1 OV and NC clones after puromycin selection. We selected one pair of these clones for further experiments. Other clones were also tested to exclude the biased conclusion due to the cloning variation. Upon examining the NR4A1 protein levels in OV cells and NC cells (Fig. 3*A*), we did not observe any significant changes in morphology between OV cells and NC cells (data not shown). However, when the OV cells and NC cells were treated with 0.5 μm TG or 0.4 mm PA, the number of NC cells decreased significantly, whereas the number of OV cells decreased more slowly. The cell survival rates detected using MTT revealed that OV cells were more viable than NC cells after treatment with TG (Fig. 3*B*) or PA (Fig. 3*C*) over the series of time points examined, and this was consistent with TUNEL assays (Fig. 3*D*).

To confirm whether NR4A1 protects against ER stress-induced apoptosis by modulating anti-apoptotic gene expression in the nucleus, we examined NR4A1 localization in OV cells after TG treatment. We treated OV cells with TG for 0, 3, or 6 h and harvested the cells to separate nuclear and cytoplasmic proteins using a nucleus and cytoplasm protein extraction kit. We confirmed the purity of the two fractions by GAPDH (a marker for cytoplasm protein) and lamin A (a marker for nucleus protein) levels in Western blotting (Fig. 3*E*). We found that in OV MIN6 cells, NR4A1 primarily localized to the nucleus upon TG treatment.

##### NR4A1 Overexpression in MIN6 Cells Reduces Caspase3 Activation upon TG or PA Treatment

**FIGURE 4.**
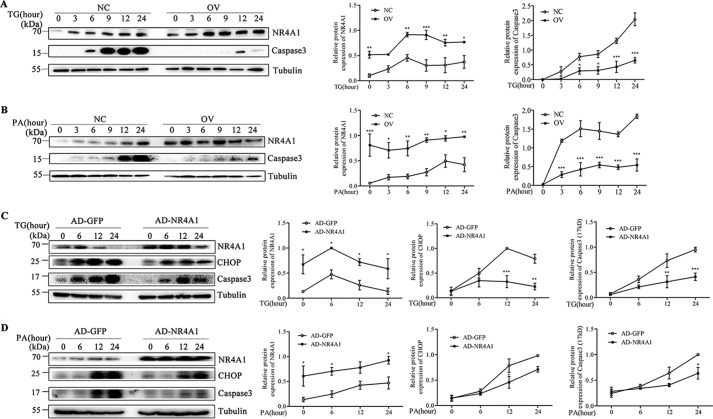
**The effect of NR4A1 on Caspase3 activation upon TG or PA treatment.** *A* and *B*, NR4A1 and active Caspase3 (17 kDa) protein levels in NC and OV cells in response to 0.5 μm TG (*A*) or 0.4 mm PA (*B*) at a series of time points. *C* and *D*, NR4A1, CHOP, and active Caspase3 (17 kDa) protein levels in response to 0.5 μm TG (*C*) or 0.4 mm PA (*D*) at a series of time points in MIN6 cell transient overexpression of NR4A1 or GFP only by adenoviral infection. Densitometric analyses of the Western blots are shown as *curves*. The data show the means of three independent experiments. *, *p* < 0.05; **, *p* < 0.01; ***, *p* < 0.001 *versus* NC.

Caspase3 activation is the canonical biological marker for apoptosis, and full-length Caspase3 is cleaved into a 17-kDa fragment during apoptosis. Therefore, we examined the levels of cleaved Caspase3 using Western blotting. We treated both NC and OV cells with TG or PA for various lengths of time, and Western blotting results revealed that OV cells had lower Caspase3 (17 kDa) levels compared with NC cells upon treatment with TG (Fig. 4*A*) or PA (Fig. 4*B*). Furthermore, Caspase3 (17 kDa) levels peaked later after TG treatment in OV cells than in NC cells. Moreover, transient overexpression NR4A1 with adenoviral infection in MIN6 cells resulted in reduced CHOP expression and decreased Caspase3 activation upon TG or PA treatment compared with control MIN6 cells infected with control adenovirus (Fig. 4, *C* and *D*).

##### NR4A1 Overexpression in MIN6 Cells Affects the UPR Profile

**FIGURE 5.**
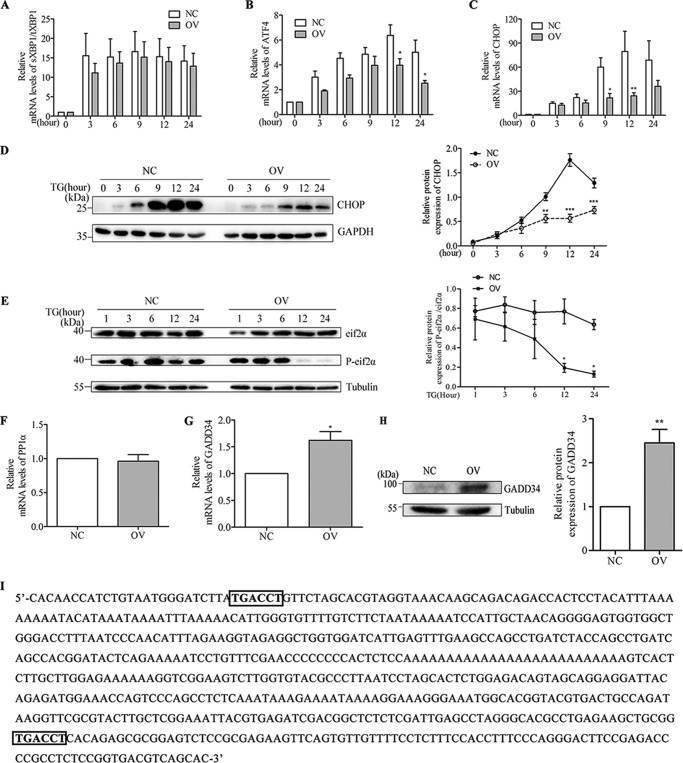
**The effect of NR4A1 overexpression on UPR.** *A* and *B*, qPCR analyses of Xbp1 mRNA splicing, shown as spliced (*s*) relative to total (*t*) (*A*) and ATF4 expression (*B*) in response to 0.5 μm TG treatment of NC and OV cells at different time points. *C* and *D*, qPCR or Western blotting analysis of CHOP in NC and OV cells in response to 0.5 μm TG treatment at a series of time points. *E*, eif2α and phosphorylated eif2α (*P-eif2*α) protein expression in response to 0.5 μm TG at a series of time points. *F* and *G*, relative mRNA levels of PP1α (*F*) and GADD34 (*G*) in NC and OV cells was assessed by qPCR. *H*, GADD34 protein levels in NC and OV cells by Western blotting. *I*, the GADD34 promoter sequence contained two putative NBREs. Densitometric analyses of the Western blots are shown as curves. The data show the means of three independent experiments. *, *p* < 0.05; **, *p* < 0.01; ***, *p* < 0.001 *versus* NC.

We treated NC and OV cells with TG and harvested the cells at various time points for RNA purification or protein extraction. qPCR results showed that there was no significant difference in XBP1 splicing between NC and OV cells (Fig. 5*A*), but activating transcription factor 4 (ATF4) and CHOP mRNA levels were decreased in OV cells compared with NC cells (Fig. 5, *B* and *C*). CHOP protein expression also decreased in OV cells upon TG treatment compared with NC cells (Fig. 5*D*). Western blotting results show that phosphorylated eif2α, a subunit of eukaryotic translation initiation factor 2α, decreased substantially in OV cells compared with NC cells at 12 h and 24 h (Fig. 5*E*).

To elucidate the mechanism by which NR4A1 expression affects PERK-CHOP signaling, we examined GADD34 and protein phosphatase 1α (PP1α) mRNA expression in OV cells and NC cells. Our data showed that NR4A1 overexpression resulted in increased GADD34 expression at both the mRNA level (Fig. 5*G*) and the protein level (Fig. 5*H*), but PP1α expression did not change (Fig. 5*F*). This difference may be explained by the presence of two putative NR4A1 binding sequences in the GADD34 promoter (Fig. 5*I*).

##### NR4A1 Overexpression Increases the mRNA and Protein Expression Levels of NF-κB, Bcl-2, and Survivin in MIN6 Cells and Mouse Islets

**FIGURE 6.**
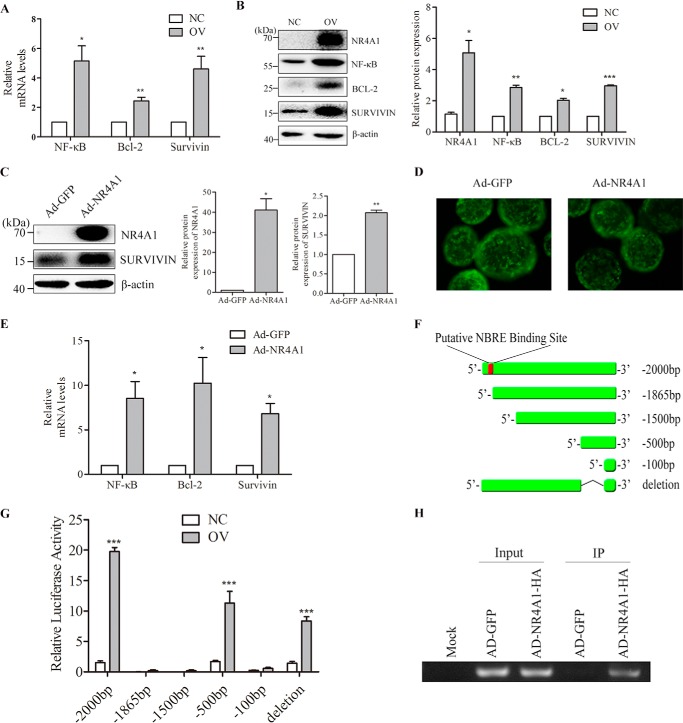
**The effect of NR4A1 overexpression on the expression of NF-κB, Bcl-2, and Survivin in MIN6 cells and mouse islets.** *A*, qPCR analysis of NF-κB, Bcl-2, and Survivin expression in NC and OV cells. *B*, NR4A1, NF-κB, Bcl-2, and Survivin protein levels in NC and OV cells were assessed by Western blotting. *C*, NR4A1 and Survivin protein expression in MIN6 cells after transient adenoviral expression. *D*, morphology of mouse islets after Ad-GFP and Ad-NR4A1 infection. Images were acquired using fluorescence microscopy. Both viruses expressed GFP. *E*, relative mRNA levels of NF-κB, Bcl-2, and Survivin in mouse islets infected with Ad-GFP or Ad-NR4A1 were determined with qPCR. Densitometric analyses of the Western blots are shown as histograms. *F*, diagram of Survivin promoters of different lengths. *G*, relative luciferase activity of Survivin promoters of different lengths in NC and OV cells. *H*, result of ChIP analysis. ChIP was used to pull down DNA fragments associated with exogenous NR4A1 with an HA tag, and a pair of primers was used to amplify the fragment of the Survivin promoter containing an NBRE binding site from the ChIP product. The data show the means of three independent experiments. *IP*, immunoprecipitate. *, *p* < 0.05; **, *p* < 0.01; ***, *p* < 0.001 *versus* NC or Ad-GFP.

The qPCR results show that NR4A1 overexpressing MIN6 cells (OV cells) had higher mRNA expression levels of NF-κB, Bcl-2, and Survivin than normal control cells (*NC*) (Fig. 6*A*). Western blotting revealed that NF-κB, BCL-2, and Survivin protein levels also increased in OV cells compared with NC cells (Fig. 6*B*). We found that transient adenoviral overexpression of NR4A1 in MIN6 cells also increased Survivin protein expression (Fig. 6*C*).

We separated and purified mouse islets and infected them with either Ad-NR4A1 or Ad-GFP. At 24 h post-infection, we observed the islets using fluorescence microscopy. The adenovirus infection rate of islets was almost 100% (Fig. 6*D*). We then performed qPCR on total RNA from the harvested islets to analyze the relative mRNA expression of NF-κB, Bcl-2, and Survivin. The mRNA levels of these three genes in the Ad-NR4A1-infected islets were increased compared with the control sample infected with Ad-GFP (Fig. 6*E*).

##### NR4A1 Enhances Survivin Promoter Transactivation via Physical Association

We cloned the NF-κB, Bcl-2, and Survivin promoters (−2000 bp to 0 bp) into the pGL3 luciferase reporter vector. NR4A1 overexpression affected Survivin promoter transactivation (Fig. 6*G*) but did not change NF-κB or Bcl-2 transactivation (data not shown).

We analyzed the Survivin promoter sequence and identified a putative nerve-growth factor inducible gene B (NGFI-B)-responsive element (NBRE) binding site sequence (TGACCT) from −1872 bp to −1866 bp. Thus, we constructed four truncated Survivin promoters (−1865 bp, −1500 bp, −500 bp, −100 bp), all of which lacked the NBRE binding site, and cloned them into pGL3 (Fig. 6*F*). The Survivin (−2000 bp) luciferase activity was higher in OV cells than in NC cells, but the other two shorter Survivin reporters (−1865 bp and −1500 bp), which lack the putative NBRE site (−1872 bp to −1866 bp), did not exhibit increased luciferase activity in OV cells compared with NC cells (Fig. 6*G*). NR4A1 overexpression did not enhance the transactivation of the shortest Survivin reporter (−100 bp) either. Interestingly, NR4A1 overexpression enhanced the activity of a shorter Survivin reporter (−500 bp) to some extent as the full-length reporter (Fig. 6*G*); therefore, we made a deletion reporter of Survivin (deleted −100 bp to −500 bp) from the full-length Survivin reporter (−2000 bp), but this reporter still contained the putative binding site, and luciferase assay data showed that NR4A1 overexpression was able to enhance the transactivation of this reporter although the enhanced transactivation was lower than that of the full-length reporter (Fig. 6*G*).

We next infected MIN6 cells with an adenovirus encoding HA-tagged NR4A1 or with a control adenovirus. The infected cells were applied for ChIP assays at 48 h post-infection. A primer pair was used to specifically amplify a region of the Survivin promoter (−1942 bp to −1711 bp) that included the NBRE sequence. A specific monoclonal anti-HA antibody was exploited for immunoprecipitation. As expected, we amplified the target fragment (232 bp) in both input samples but not in the mock sample (Fig. 6*H*). This target fragment was also successfully amplified in the pulldown from the Ad-NR4A1-HA-infected MIN6 cells but not from the control Ad-GFP-infected MIN6 cells.

##### NR4A1 Knockdown Affects the Expression of Anti-apoptotic and Pro-apoptotic Genes

**FIGURE 7.**
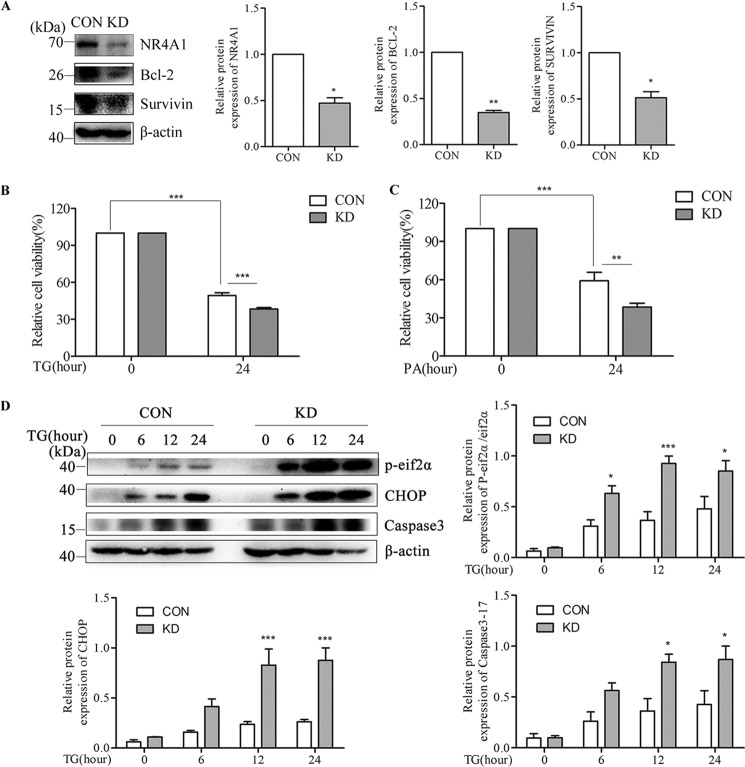
**The effects of NR4A1 knockdown on the expression of Survivin and Bcl-2 and on ER stress-induced apoptosis in MIN6 cells.** Analyses of MIN6 cell stably knockdown NR4A1 clone (designated as KD) and normal control clone (designated as CON). *A*, protein expression of NR4A1, Bcl-2, and Survivin in CON and KD cells. *B* and *C*, the cell viability of CON and KD cells treated with TG (*B*) or PA (*C*) for 24 h was analyzed with an MTT assay. *D*, changes in protein expression of NR4A1, phosphorylated eif2α (*P-eif2*α), CHOP, and Caspase3 (17 kDa) in CON and KD cells treated with 0.5 μm TG. Densitometric analyses of the Western blots are shown as histograms or curves. The data show the means of three independent experiments. *, *p* < 0.05; **, *p* < 0.01; ***, *p* < 0.001 *versus* CON.

To further confirm the above observations, we infected MIN6 cells with a lentivirus encoding siRNA against mouse NR4A1 or with a control lentivirus and selected cell clones using puromycin. We obtained several NR4A1 knockdown clones (*KD*) in which the NR4A1 expression level was <70% that in control cells (*CON*), and chose one pair of them as representation (Fig. 7*A*). Other clones were also tested to exclude false conclusion due to cloning variation. KD cells also exhibited reduced Survivin and Bcl-2 protein expression compared with CON cells (Fig. 7*A*). After TG or PA treatment of CON and KD cells, MTT results show that the viability of KD cells was reduced compared with CON cells (Fig. 7, *B* and *C*). Western blotting results showed that eif2α phosphorylation or activation increased in KD cells upon TG treatment, TG-induced CHOP protein expression was much higher in KD cells, and TG-induced active Caspase3 (17 kDa) was also increased in KD cells compared with CON cells (Fig. 7*D*).

##### NR4A1 Expression in Mouse Islets Affects Apoptosis Occurrence Induced by TG or PA

**FIGURE 8.**
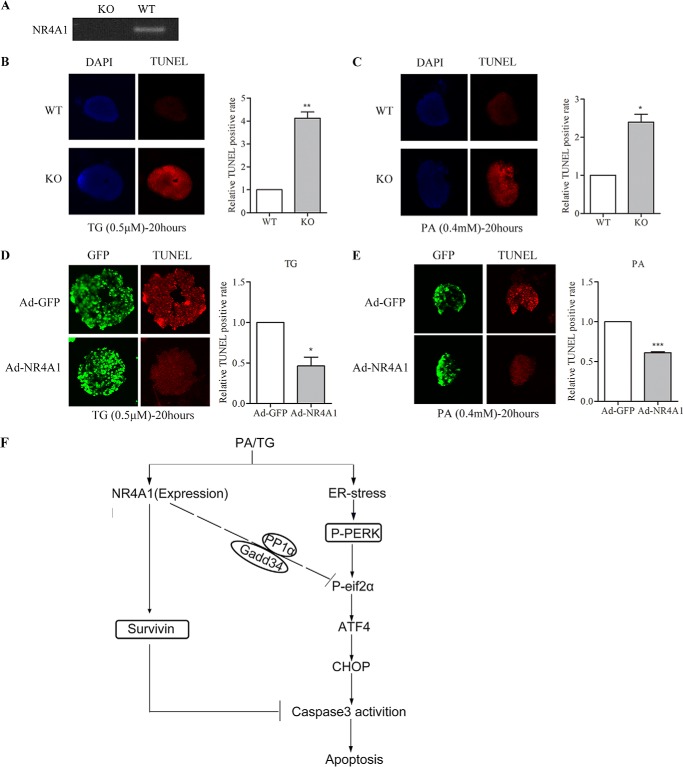
**Mouse islets expressing different NR4A1 levels determines their ability to resist TG- or PA-induced apoptosis.** *A*, genotype confirmation of global NR4A1 knock-out mice (KO) and wild-type mice (WT) using RT-PCR to analyze their islets. *B* and *C*, TUNEL assay of islet cells from KO and WT mice treated with 0.5 μm TG (*B*) or o.4 mm PA (*C*) for 20 h. *Blue* represents DAPI, and *red* represents the apoptotic cells. *D* and *E*, TUNEL assay of islet cells from wild-type mice infected with Ad-GFP or Ad-NR4A1 after treated with 0.5 μm TG (*D*) or o.4 mm PA (*E*) for 20 h. *Green* represents GFP, and *red* represents the apoptotic cells. The histograms were made to assess relative fluorescence intensity (=total red densitometry value/islet surface area). The data show the means of three independent experiments; *, *p* < 0.05; **, *p* < 0.01; ***, *p* < 0.001 *versus* WT or Ad-GFP. *F*, a model for possible roles of NR4A1 in protecting ER stress induced pancreatic β-cell apoptosis.

To investigate whether a lack of NR4A1 effects β-cell death under ER stress conditions in mouse islets, we purified islets from global NR4A1 knock-out mice (*KO*) and wild-type mice (*WT*). First, we did genotype identification by RT-PCR to amplify the full-length cDNA using a specific pair of primer and found there is no mRNA expression of NR4A1 in KO mice islets compared with WT mice (Fig. 8*A*). We treated the purified islets with TG or PA for 20 h, and TUNEL assays showed that the rate of apoptotic cells is much higher in KO islets than that in WT (Fig. 8, *B* and *C*). Moreover, we also overexpressed NR4A1 in islets from wild-type mice by viral infection with Ad-NR4A1. After infection for 48 h, the islets were treated with TG or PA for 20 h, and TUNEL assays showed that the rate of apoptotic cells is much lower in NR4A1 overexpressed islets than that of control islets infected with control virus (Ad- GFP) (Fig. 8, *D* and *E*).

## Discussion

As an early response factor or anti-stress factor, the ER stress inducers TG and free fatty acids could induce NR4A1 mRNA expression in various cell types as previously reported (8, 9). We confirmed that conclusion in MIN6 cells and additionally checked NR4A1 protein expression. We found TG was able to induce NR4A1 expression in β-cells of mouse islets; PA had the same effect (data not shown). Approximately 80% of the cells in the mouse islet are pancreatic β-cells; thus, our data from islet cells likely reflect the behavior of pancreatic β-cells. We found that TG-and PA-induced NR4A1 expression peaked earlier than Bip (immunoglobulin-binding protein), and CHOP but was similar to XBP1 splicing in islets. Therefore, ER stress inducers such as TG and PA induce NR4A1 expression in parallel with earlier UPR molecules in mouse islets, whereas this induced NR4A1 expression is not the consequence of UPR downstream signaling.

It is not known if NR4A1 as an anti-stress protein has the role to resist ER stress-induced β-cell apoptosis. Our data showed that overexpression NR4A1 in MIN6 cells reduced the cell death rate upon TG or PA treatment and knocking-down NR4A1 in MIN6 cells increased the cell death rate. Our TUNEL assays exhibited TG- or PA-induced β cell apoptosis, which was further confirmed by Western blotting using a specific antibody to recognize 17-kDa Caspase3 (the active form of Caspase3). We found overexpression NR4A1 in mice islets was able to resist TG- or PA-induced cell apoptosis, whereas the islets from NR4A1 knock-out mice were much more sensitive to TG- or PA-induced cell apoptosis.

To decipher the related mechanism of the protection effect of NR4A1 on β-cells, we found overexpression of NR4A1 in MIN6 cells reduced the formation of 17-kDa Caspase3 and decreased protein expression of CHOP, whereas knocking-down NR4A1 expression exhibited the opposite trend. It is known that sustained UPR or ER stress leads to CHOP protein expression and that CHOP overexpression results in Caspase3 activation, which is a critical step in apoptosis.

There are three branches of the UPR signaling pathways, the downstream signaling of three UPR sensors, IRE1, ATF6, and PERK, on the cytosolic ER membrane (27). Among these UPR signaling pathways, ER stress results in IRE1 phosphorylation, which triggers downstream signaling and XBP1 splicing. The final results of this signaling are to generate new chaperones and to degrade unfolded proteins (28, 29). Furthermore, activated IRE1 degrades mRNA, thus reducing protein synthesis. Therefore, this signaling pathway is beneficial for the ER to release its stress or burden (30, 31). ER stress also results in PERK phosphorylation or PERK activation, which induces eif2α phosphorylation (activation). Eif2α activation activates ATF4, which indirectly induces downstream CHOP expression (32, 33). Mild PERK activation releases ER stress through a negative feedback loop, but strong and sustained PERK activation generates excess CHOP and results in apoptosis (34, 35). It is important to clarify how NR4A1 regulates CHOP activation. We found that NR4A1 overexpression in MIN6 cells did not change the XBP1 splicing profile (sXBP1) upon TG treatment but changed the eif2α phosphorylation profile significantly at certain time points and reduced the mRNA expression of ATF4 and CHOP. Knocking-down NR4A1 expression in MIN6 cells also increased TG-induced eif2α phosphorylation, CHOP expression, and 17-kDa caspase3 formation. These data suggest that NR4A1 expression level has the impact on PERK downstream signaling in MIN6 cells upon TG treatment, especially the CHOP expression. We found overexpression NR4A1 did not change PP1α expression but increased GADD34 expression. We examined the GADD34 promoter sequence and identified two putative NR4A1 (or NBRE) binding sites in the promoter. PP1α is a reported eif2α phosphatase, and GADD34 is a PP1α association protein or activator (36, 37) so we predict that NR4A1 modulates eif2α phosphorylation by enhancing GADD34 expression. The mechanism by which NR4A1 modulates PERK/eif2α/ATF4/CHOP signaling requires further study.

Besides the pro-apoptosis genes, we also found that overexpression of NR4A1 resulted in increased expression of NF-κB, Bcl-2, and Survivin at mRNA or protein levels in MIN6 cells or purified mouse islets, and knocking-down NR4A1 expression in MIN6 cells resulted in decreased expression of these three genes. It has been reported that Nur77 (NR4A1) inhibits apoptosis in HEK293 cells through NF-κB activation (38). Another report showed that NF-κB enhances Bcl-2 expression (39). It has been reported that Survivin is a direct inhibitor of Caspase3. It has been documented that Survivin specifically binds to pro-caspase3 and inhibits Caspase3 activation and reduces cell death induced by diverse stimuli (40–42). Therefore, Survivin is an inhibitor of apoptosis.

We performed reporter assays to elucidate the mechanism by which NR4A1 modulates NF-κB, Bcl-2, and Survivin expression. We found overexpression NR4A1 did not increase NF-κB or Bcl-2 reporter transactivation; we analyzed the NF-κB or Bcl-2 promoter sequences but did not find a putative NBRE site in these sequences. We predict that NR4A1 indirectly modulates NF-κB and Bcl-2 expression, which needs further investigation. We found that NR4A1 overexpression increased the transactivation of Survivin reporter with a promoter length of 2000 bp but failed to enhance the transactivation of the two shorter truncated Survivin promoters (−1865 bp and −1500 bp), which lack the putative NBRE site (−1872 bp to 1866 bp). Our ChIP assay further confirmed that NR4A1 has a physical association with the Survivin promoter including the putative NR4A1 binding site.

These data indicate that there is a critical NBRE site (43) in the Survivin promoter for NR4A1 to regulate Survivin expression. It has previously been reported that NR4A1 enhances Survivin transactivation by exploiting two reporters with two short promoter regions of Survivin (−150 bp and −269 bp) (44). We also observed that NR4A1 enhanced the Survivin reporter activation with a shorter promoter region (−500 bp), which is consistent with those previous reports (44). We examined the sequence of this promoter region (−500 bp to 0 bp) and did not find a putative NR4A1 binding site; therefore, we conclude that NR4A1 does not directly bind to this region and that the enhanced transactivation may be due to indirect modulation. There is likely a repressor that associates with the Survivin promoter regulatory element between −1865 bp and −500 bp. We further observed two Survivin reporters and found the shortest reporter (−100 bp) lost the transactivation enhancement by NR4A1, and the deletion reporter (from −2000 bp, lack −100 to −500 bp) had the enhanced transactivation by NR4A1 due to the NR4A1 binding site.

It has been reported that under some drug treatments, NR4A1 translocates from the nucleus to the cytoplasm and induces a conformational change in Bcl-2 to trigger cell apoptosis (18, 45). Therefore, NR4A1 localization determines its function as a pro-apoptotic factor or an anti-apoptotic factor. Our data show that overexpressed NR4A1 primarily localized to the nucleus in MIN6 cells during TG treatment. This result indicates that the protective effect of NR4A1 against TG-induced apoptosis in MIN6 cells is likely related to its function as a transcription factor.

In summary, ER stress inducers such as TG or PA induce the expression of NR4A1 in a pancreatic β-cell line or in purified mouse islets. NR4A1 overexpression in MIN6 cells confers resistance to TG- or PA-induced apoptosis, whereas NR4A1 knockdown in MIN6 cells results in increased apoptosis upon TG or PA treatment. NR4A1 protects pancreatic β-cells from ER stress-mediated apoptosis by directly up-regulating the expression of Survivin (an anti-apoptotic protein) and indirectly down-regulating the expression of CHOP, which we term “positive and negative regulation.”

## Author Contributions

C. Y. conducted the research and the data analysis. S. C., D. Q., P. G., and C. C. performed mice islets study. Y. L. helped in data analysis. W. G., T. X., and C. Z. helped in conducting research. Q. G. and Y. F. helped in the conception and design of the experiments. X. L. helped in designing the experiments and supervising the integrity of the data and the accuracy of the data analysis. X. W. designed the research and wrote paper. All authors read and approved the final manuscript.

## Footnotes

3 The abbreviations used are:

ER: endoplasmic reticulum
UPR: unfolded protein response
TG: thapsigargin
MTT: 3-(4,5-dimethylthiazol-2-yl)-2,5-diphenyltetrazolium bromide
OV: overexpression
NC: normal control
CON: control
PA: palmitic acid
qPCR: quantitative real-time PCR
CHOP: C/EBP homologous protein
ATF4: activating transcription factor 4
GADD34: growth arrest and DNA damage-inducible protein-34
PP1α: protein phosphatase 1α
NBRE: nerve-growth factor inducible gene B (NGFI-B)-responsive element.
